# Transverse aortic constriction induces gut barrier alterations, microbiota remodeling and systemic inflammation

**DOI:** 10.1038/s41598-021-86651-y

**Published:** 2021-04-01

**Authors:** Nicola Boccella, Roberta Paolillo, Lorena Coretti, Stefania D’Apice, Adriano Lama, Giuseppe Giugliano, Gabriele Giacomo Schiattarella, Mariella Cuomo, Ilaria d’Aquino, Gina Cavaliere, Orlando Paciello, Maria Pina Mollica, Giuseppina Mattace Raso, Giovanni Esposito, Francesca Lembo, Cinzia Perrino

**Affiliations:** 1 Department of Advanced Biomedical Sciences, Federico II University, Via Pansini 5, 80131 Naples, Italy; 2 Fondazione Umberto Veronesi, Milan, Italy; 3 Department of Pharmacy, Federico II University, Via D. Montesano 49, 80131 Naples, Italy; 4 Department of Physiology and Biochemistry, Faculty of Medicine and Surgery, University of Malta, Msida, Malta; 5 Task Force On Microbiome Studies, Federico II University, Naples, Italy; 6 Department of Molecular Medicine and Medical Biotechnology, Federico II University, Naples, Italy; 7 Department of Veterinary Medicine and Animal Productions, Unit of Pathology, Federico II University, Naples, Italy; 8 Department of Biology, Federico II University, Naples, Italy

**Keywords:** Cardiac hypertrophy, Microbiome

## Abstract

Accumulating evidence suggests that modifications of gut function and microbiota composition might play a pivotal role in the pathophysiology of several cardiovascular diseases, including heart failure (HF). In this study we systematically analysed gut microbiota composition, intestinal barrier integrity, intestinal and serum cytokines and serum endotoxin levels in C57BL/6 mice undergoing pressure overload by transverse aortic constriction (TAC) for 1 and 4 weeks. Compared to sham-operated animals, TAC induced prompt and strong weakening of intestinal barrier integrity, long-lasting decrease of colon anti-inflammatory cytokine levels, significant increases of serum levels of bacterial lipopolysaccharide and proinflammatory cytokines. TAC also exerted effects on microbiota composition, inducing significant differences in bacterial genera inside Actinobacteria, Firmicutes, Proteobacteria and TM7 phyla as shown by 16S rDNA sequencing of fecal samples from TAC or sham mice. These results suggest that gut modifications represent an important element to be considered in the development and progression of cardiac dysfunction in response to TAC and support this animal model as a valuable tool to establish the role and mechanisms of gut-heart crosstalk in HF. Evidence arising in this field might identify new treatment options targeting gut integrity and microbiota components to face adverse cardiac events.

## Introduction

Mammalian gut microbiota is composed by a diverse selection of microorganisms colonizing the gastrointestinal tract, generating a complex ecosystem with remarkable effects on nutrition, gut epithelial cell health, immunity, and inflammation^1^. Different variables can affect microbiota composition, including genetics, age, diet, environmental factors and several human diseases^2,3^. Interestingly, various experimental strategies have been described to modulate gut microbiota composition, including administration of antibiotics, probiotics, prebiotics, postbiotics, and fecal microbiota transplantation, associated to temporary or prolonged restoration of gut function, microbiota composition and immune response^4^.

Several cardiovascular diseases (CVD) have been commonly associated with remarkable changes in gut barrier function and microbiota composition through different, still largely undefined mechanisms, presumably acting at multiple levels^1,5^. A mutual gut-heart crosstalk has been recently proposed in heart failure (HF), even if messengers and underlying mechanisms are still not entirely defined^6^. Reduced cardiac output and peripheral vasoconstriction in HF can induce intestinal hypoperfusion, disrupt intestinal barrier function, promote systemic inflammation and affect gut microbiota composition^6–8^. The potential roles of specific bacteria in the pathogenesis of cardiometabolic disorders and their therapeutic implications are now starting to be elucidated^9–11^. In addition, gut microbiota-derived molecules, either structural components or bioactive products, can exert remote effects through the activation of different signaling pathways in CVD^7,12,13^. In this context, despite species-specific limitations, experimental systems including animal models are crucial to test for causal connections and provide novel insight into host–microbiota interactions modeling human health and diseases.

The murine model of transverse aortic constriction (TAC) is one of the most well-established and widely used preclinical models of pressure overload-induced cardiac hypertrophy and failure^14–16^. It has been recently demonstrated that choline diet and its gut microbe-derived metabolite Trimethylamine N-Oxide (TMAO) exacerbate TAC-induced HF, while nonlethal inhibition of TMAO production improves cardiac function and remodeling after TAC, clearly indicating that gut microbiota can affect cardiac function and remodeling induced by pressure overload^17,18^. In the present study, we investigated the effects of TAC on intestinal barrier integrity, intestinal and serum cytokines, serum endotoxin levels and gut microbiota composition in C57BL/6 mice. Our results suggest that gut modifications might represent an important variable in the development and progression of cardiac dysfunction in response to TAC, and support this murine model as a valuable tool to establish the role and mechanisms of gut-heart crosstalk in HF.

## Results

### Intestinal permeability upon TAC leads to systemic LPS translocation and inflammation

**Figure 1:**
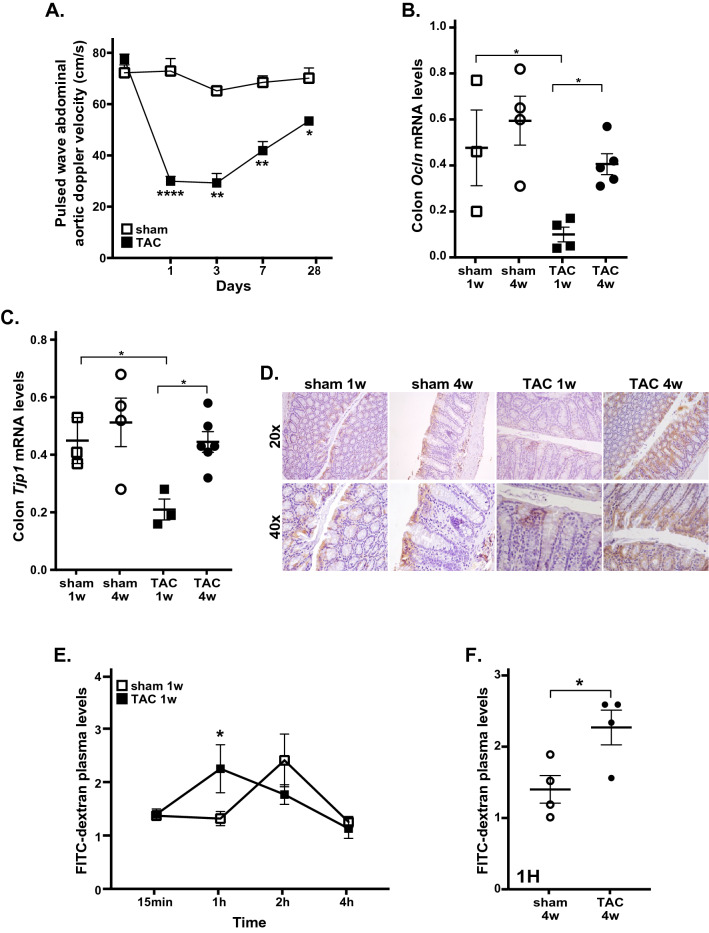
Effects of TAC or sham surgery on intestinal barrier integrity. (**A**) Abdominal aortic blood flow (cm/s) was evaluated at 1, 3, 7 and 28 days after surgical procedure in sham and TAC mice by Pulsed Wave Doppler (sham n = 6; TAC n = 10). (**B**,**C**) mRNA expression levels of occludin (*Ocln,* **B**) and tight junction protein ZO-1 (*Tjp1,* **C**) in colon samples from sham or TAC mice (sham: n = 3–4; TAC: n = 3–6). (**D**) Representative images of immunohistochemical analysis of tight junction protein ZO-1 in colon samples from the different groups of mice (sham 1w: n = 6; sham 4w: n = 5; TAC 1w: n = 4; TAC 4w: n = 6). ZO-1 positive cells were stained in brown; bigger brown and deeper color represent higher ZO-1 protein levels. Pictures are shown at 20 × and 40 × magnification. (**E**) Plasma levels of FITC-dextran 4000 at 15 min, 1, 2, 4 h after gavage in sham 1w and TAC 1w mice (sham 1w: n = 14; TAC 1w: n = 12). (**F**) Plasma levels of FITC-dextran 4000 1 h after gavage in sham 4w and TAC 4w (sham 4w: n = 4; TAC 4w: n = 4). Results are presented as mean ± SEM. Statistical significances were assessed using one-way ANOVA followed by Newman-Keuls multiple comparison post-hoc test (**A**) or Tukey’s comparison test as appropriate (**B**–**F)**.

As expected, TAC induced left ventricular hypertrophy and systolic dysfunction (left ventricle weight/body weight ratio: sham 1w = 3.5 ± 0.1, TAC 1w = 5.4 ± 0.5*, sham 4w = 3.4 ± 0.1, TAC 4w = 5.2 ± 0.4*; % fractional shortening: sham 1w = 59.1 ± 0.5, TAC 1w = 51.8 ± 0.9, sham 4w = 59.8 ± 0.3, TAC 4w = 46.2 ± 1.9*; *p < 0.01 vs. sham, Table 1). Immediately after TAC, abdominal aortic blood flow was significantly reduced in TAC mice compared to sham, resulting in intestinal hypoperfusion (Fig. 1A). Consistently, colon HIF 1-α levels were significantly increased after TAC 1w, and recovered in TAC 4w mice (Supplementary Fig. 1). Decreased intestinal perfusion in TAC 1w mice was associated to a prompt and strong weakening of intestinal barrier, as shown by reduced mRNA expression of *Ocln* and *Tjp1* (Fig. 1B,C; see Supplementary Fig. 2 for pre-surgery values), and reduced immunostaining of zonula occludens-1 (zo-1) after 1w of pressure overload (Fig. 1D).

**Table 1:** Cardiac morphometry and echocardiography of mice from the different groups.

Morphometry	Sham 1w	TAC 1w	Sham 4w	TAC 4w
	(n = 4)	(n = 4)	(n = 15)	(n = 18)
BW, g	23.9 ± 0.6	23.7 ± 0.6	26.1 ± 0.7	24.9 ± 0.6
LVW, mg	80.2 ± 1.5	114.7 ± 8.1*	88.4 ± 3.3	149.0 ± 6.4*^#^
HW, mg	110.0 ± 2.7	150.1 ± 10.2*	124.1 ± 4.7	193.3 ± 8.4*^#^
LVW/BW, mg/g	3.5 ± 0.1	5.4 ± 0.5*	3.4 ± 0.1	5.2 ± 0.4*
HW/BW, mg/g	4.8 ± 0.1	7.0 ± 0.7*	4.8 ± 0.2	7.8 ± 0.3*

Echocardiography	Sham 1w	TAC 1w	Sham 4w	TAC 4w
	(n = 20)	(n = 25)	(n = 15)	(n = 18)
LVEDd, mm	3.3 ± 0.1	3.2 ± 0.05	3.4 ± 0.1	3.3 ± 0.1
LVESd, mm	1.4 ± 0.1	1.5 ± 0.04*	1.4 ± 0.1	1.8 ± 0.1*
IVSd, mm	0.9 ± 0.1	1.0 ± 0.02*	0.9 ± 0.1	1.2 ± 0.1*^#^
PWd, mm	0.9 ± 0.1	1.1 ± 0.04*	0.9 ± 0.1	1.3 ± 0.04*^#^
FS, %	59.1 ± 0.5	51.8 ± 0.9*	59.8 ± 0.3	46.2 ± 1.9*
EF, %	89.7 ± 0.4	83.6 ± 0.9*	90.0 ± 0.2	77.8 ± 2.6*^#^
HR, bpm	652 ± 8.4	635 ± 11	617 ± 14	655 ± 9.8

Data are presented as mean ± SEM. An unpaired Student’s t-test was performed for each pair of four groups and subsequent multiple comparisons were made with use of the Tukey method. A p value < 0.05 was considered significant (*p < 0.05 vs. correspondent sham; ^#^p < 0.05 vs. correspondent TAC 1w).

*BW* Body weight,* LVW* left ventricle weight, *HW* heart weight,* LVEDd* left ventricular end-diastolic diameter,* LVESd* left ventricular end-systolic diameter,* IVSd* interventricular septum end-diastolic diameter,* PWd* posterior wall end-diastolic diameter, *FS* fractional shortening, *EF* ejection fraction, *HR* heart rate.

To determine the effects of TAC on gut barrier function, we analyzed circulating levels of FITC-dextran D4000 at different time intervals after oral administration by gavage in sham 1w and TAC 1w mice. In TAC 1w mice, circulating levels of FITC-dextran significantly increased 1 h after oral gavage, and thereafter decreased. In contrast, time-course of FITC-dextran circulating levels was delayed in sham 1w mice compared to TAC 1w, reaching peak concentration 2 h after oral administration, and decreasing thereafter (Fig. 1E). Gut barrier function was still functionally impaired in TAC 4w mice, as shown by differences in circulating levels of FITC-dextran between TAC 4w and sham 4w mice 1 h after gavage (Fig. 1F).

**Figure 2:**
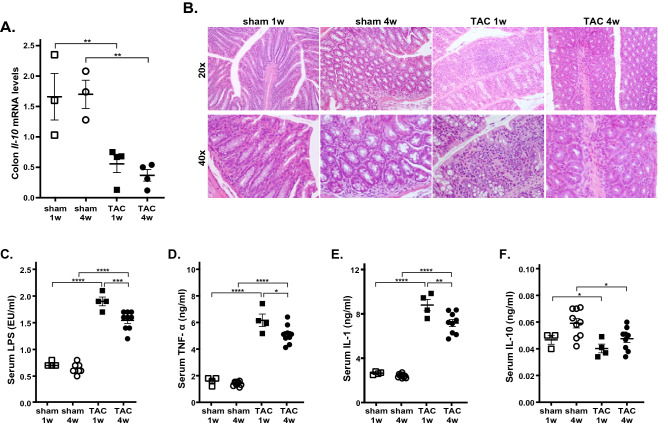
Effects of TAC or sham surgery on inflammation. (**A**) mRNA expression levels of Interleukin-10 (*Il10*) in colon samples (sham: n = 3–4; TAC: n = 3–6). (**B**) Representative hematoxylin and eosin–stained sections from colon tissues of mice at original magnifications ×20 and ×40. Histological evaluation of inflammatory cells infiltration was scored along the entire colon length, inspecting the colon mucosa, submucosa and transmural areas considering the following parameters: (a) severity of inflammatory cell infiltration (sham 1w = 0.83 ± 0.16, sham 4w = 1.40 ± 0.24, TAC 1w = 2.25 ± 0.25, TAC 4w = 1.83 ± 0.31); (b) extent of the inflammation as expansion of leukocyte infiltration (sham 1w = 1.17 ± 0.31, sham 4w = 1.20 ± 20, TAC 1w = 2 ± 0, TAC 4w = 1.50 ± 0.22); and (c) presence of fibrosis (sham 1w = 0 ± 0, sham 4w = 0 ± 0, TAC 1w = 0.25 ± 0.25, TAC 4w = 0.33 ± 0.21). For details on the histological scoring system please see Materials and Methods section. Data reported are mean ± SEM (sham 1w: n = 6; sham 4w: n = 5; TAC 1w: n = 4; TAC 4w: n = 6). Serum levels of (**C**) lipopolysaccharide (LPS), (**D**) tumor necrosis factor-α (TNF-α), (**E**) interleukin-1 (IL-1) and (**F**) IL-10 in all experimental groups (sham: n = 4–8; TAC: n = 4–9). Results are presented as mean ± SEM. Statistical significances were assessed using one-way ANOVA followed by Newman–Keuls multiple comparison post-hoc test (**A**) or Tukey’s comparison test as appropriate (**B**–**F**).

Colon expression levels of anti-inflammatory cytokine interleukin-10 (IL-10) were significantly reduced in TAC 1 w and TAC 4w colon samples compared to respective sham (Fig. 2A; see Supplementary Fig. 2 for pre-surgery values). These changes were associated to the histological evidence of remarkable inflammatory infiltrate in murine colon samples from TAC 1w mice (Fig. 2B). Consistent with these results, serum levels of lipopolysaccharide (LPS) and proinflammatory cytokines tumor necrosis factor-α (TNF-α) and interleukin-1 (IL-1), were rapidly and persistently enhanced after TAC surgery (Fig. 2C), while circulating levels of IL-10 were reduced in TAC mice (Fig. 2C–F).

### TAC impacts on fecal microbiota composition

**Figure 3:**
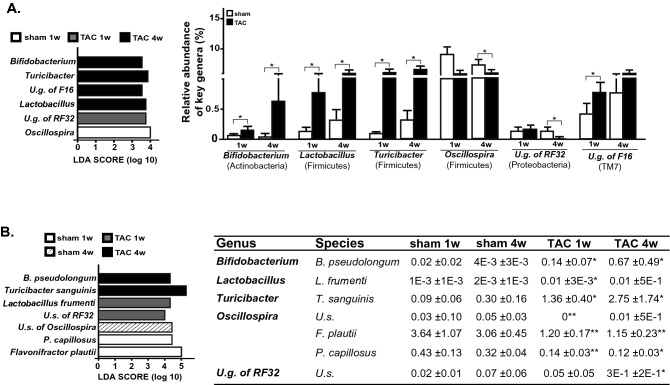
Gut microbiota composition after sham or TAC surgery in mice. Gut microbiota differences based on 16S rDNA sequencing at genus (**A**) and species (**B**) taxonomic levels were identified using linear discriminant analysis (LDA) combined with effect size (LEfSe) algorithm. In each panel, LDA scores (left) and relative abundance (right) of key phylotypes discriminating sham and TAC bacterial communities are reported (sham n = 8; TAC n = 9). Statistical significances were assessed using LEfSe analysis with alpha values of 0.05 for both Kruskal–Wallis and pairwise Wilcoxon tests and a cutoff value of LDA score (log10) above 2.0 (*p < 0.05 and **p < 0.01 vs. correspondent sham).

Gut barrier integrity is closely linked to gut microbiota composition, and they can be reciprocally affected, especially in response to external pathological causes. Differences in fecal microbiota composition among sham and TAC mice were determined by 16S rDNA sequencing and a Good’s coverage index > 99% was obtained at the rarefaction point of 26,396 reads/sample. Comparison of fecal gut microbiota communities among groups revealed significant changes of bacterial genera inside Actinobacteria, Firmicutes, Proteobacteria and TM7 phyla. Specifically, intergroup differences at genus and species levels were analyzed by the linear discriminant analysis (LDA) effect size (LEfSe), identifying the genera *Bifidobacterium*, *Lactobacillus*, *Turicibacter*, unclassified genus (u.g.) of RF32 and u.g. of F16 as characteristic of TAC mice, whereas the genus *Oscillospira* was significantly less abundant in TAC mice compared to sham at specific time windows (Fig. 3A).

SPINGO high-resolution approach was used to obtain bacterial species assignment of key genera with significant differences among groups (Fig. 3B). After TAC, a significant increase in OTUs resembling *Bifidobacterium pseudolongum, Turicibacter sanguinis, Lactobacillus frumenti*, and an unclassified species belonging to Proteobacteria phylum (order RF32) was detected, along with decrease of species within *Oscillospira* genus (*Pseudoflavonifractor capillosus* and *Flavonifractor plautii*, Fig. 3B).

In order to predict functional effects of gut microbiota alterations induced by TAC, we performed a Phylogenetic Investigation of Communities by Reconstruction of Unobserved States (PICRUST) analysis. This analysis revealed a significant increase in pathways inducing L-lactate dehydrogenase after 1w TAC (pre-surgery: 2,445.6 ± 272.9; sham 1w: 2,038.4 ± 323.9; TAC 1w: 3,074 ± 331.6; p < 0.05 for sham 1w vs. TAC 1w according to two-tailed nonparametric Kruskal–Wallis test). Conversely, a trend to decrease of KEGG (Kyoto Encyclopedia of Genes and Genomes) functions involved in butyrate production (butyryl-CoA dehydrogenase, phosphate butyryltransferase, butyrate kinase) was observed after 4w TAC (data not shown). Collectively, these data indicate that a significant remodeling of specific bacterial species abundance within identified key genera occurs soon after TAC, identifying a clear effect of the surgery on microbiota profiles and, possibly, on microbiota functionality.

## Discussion

In the present study we demonstrate that the murine model of TAC induces prompt and strong weakening of intestinal barrier integrity, decrease of colon anti-inflammatory cytokine levels, increase of serum levels of LPS and proinflammatory cytokines, and significant differences in fecal bacterial genera inside Actinobacteria, Firmicutes, Proteobacteria and TM7 phyla. Our findings clearly support this murine model as a valuable tool to establish the importance of gut barrier function and microbiota composition in HF.

Several studies have demonstrated that a decrease in gut perfusion structurally and functionally affects intestinal barrier integrity^19^. In patients with HF, both alteration of villi shape in intestinal mucosa resulting in intramucosal acidosis^20,21^ and intestinal wall thickness with edema have been demonstrated^22^. Moreover, even if with multiple discrepancies, alterations in gut microbial communities have been reported in patients with HF^22–29^. Our experiments showed that TAC was associated with a prompt reduction in abdominal aortic blood flow and intestinal hypoperfusion, accompanied by a rapid impairing of gut barrier structure as demonstrated by reduction of zonula occludens-1 and occludin levels and increased FITC-dextran translocation across intestinal epithelium into blood. Moreover, we concurrently found a significant increase of LPS and proinflammatory cytokines in systemic blood circulation of TAC mice compared to sham. Of note, together with remarkable inflammatory infiltrate persistence and high levels of inflammatory markers, gut barrier was still functionally compromised in TAC 4w mice, as revealed by FITC-dextran in vivo permeability assay, despite a recovery of tight junctions mRNA and proteins levels at this time point. We cannot exclude that at later time points gut barrier function could be restored.

These dramatic alterations of gut barrier due to hypoperfusion induced by TAC were associated with remarkable changes in gut microbiota composition. Based on LEfSE results, taxa correlated to dysbiosis and colonic inflammation, such as F16, significantly increased after TAC^30–32^. Furthermore, we identified unclassified genus of RF32 (Proteobacteria phylum) as a potential microbial biomarker and source of LPS in TAC operated mice, possibly contributing to colonic inflammation. Moreover, in TAC 4w mice gut microbiota was characterized by an increase in genera *Turicibacter and Lactobacillus*. The increase of these lactate-producing bacteria (namely *T. sanguinis* and *L. frumenti*, according to species-level SPINGO classification) concurred with the depletion of butyrate-producing bacteria (genus *Oscillospira*, taxonomically classified as *P. capillosus* and *F. plautii* species after SPINGO procedure) in TAC mice, and these results were also corroborated by PICRUST analysis. Recent studies have implicated increases in genus *Lactobacillus* in pathophysiology of cardiovascular diseases, even if conflicting results have been reported in HF. Increases in lactate-producing *Lactobacillus* have been demonstrated in elderly patients with HF and in animal models of hypertension^24,33^. Consistently, ST-segment elevation myocardial infarction (STEMI) patients were characterized by increased circulating levels of intestinal Lactobacilli, associated with systemic inflammation and adverse cardiovascular events^33^. On the contrary, ferulic acid administration, which improves cardiac function in TAC mice, has been shown to increase intestinal *Lactobacillus*^34^. Moreover, a reduction of *Lactobacillus* has been reported in gut microbiota of rats with isoproterenol-induced HF^35^. Thus, further investigations will be necessary to clarify the role of *Lactobacillus* phylotypes in the pathophysiology of HF, ideally with species-level approaches.

A significant depletion of genus *Oscillospira*, an under-investigated bacterium usually considered a biomarker of intestinal and host wellness, was also identified in TAC mice. Presence of this bacterium has been associated to leanness, resulting depleted in obese patients and reduced in diseases that involve inflammation, and these associations have been inferred to the putative capacity of *Oscillospira* species to produce butyrate^36^. Butyrate-producing bacteria are considered relevant colonizers of the gastrointestinal tract with known anti-inflammatory effects and a prominent role in maintaining intestinal barrier integrity^37,38^. Consistent with our findings in TAC mice, butyrate-producing bacteria such as *Faecalibacterium* and *Blautia* genera, were also depleted in other animal models of hypertension^39^ and in studies involving patients with HF^24–26^. Thus, the unbalance between butyrate and lactate producing bacteria might represent a possible mark of gut microbiota adaptations to the new environment induced by intestinal hypoperfusion in presence of HF. Overall, we propose that gut hypoperfusion induced by TAC structurally and functionally affects intestinal barrier with an effect on the balance of gut microbiota composition. TAC-induced gut dysbiosis and enhanced gut permeability may in turn affect systemic inflammation possibly contributing to cardiac dysfunction.

## Conclusions

Alterations of gut structure/function and dysbiosis may represent important elements to be considered in the development or progression of cardiac dysfunction in response to pressure overload induced by TAC. Whether restoration of gut function and microbiota composition might exert a beneficial effect on cardiac remodeling and dysfunction is still unknown and will require further investigations. However, our findings clearly support the murine model of TAC as a valuable tool to establish the importance of gut barrier function and microbiota composition in HF, suggesting novel important avenues of research in this field, including administration of single or defined cocktails of bacterial species to counteract alterations in gut barrier integrity during HF.

## Methods

### Ethics statement

All experiments and methods were performed in accordance with relevant guidelines and regulations. All experiments involving animals in this study were conform to the *Guide for the Care and Use of Laboratory Animals* published by the US National Institutes of Health. All in vivo experimental protocols were approved by the animal welfare regulation of University of Naples “Federico II”, Italy, and by the Superior Institute of Health, Italy. Animal studies were carried out in compliance with the ARRIVE (Animal Research: Reporting of In Vivo Experiments) guidelines.

### Experimental animals

C57BL/6 (Charles River Laboratories) mice of either sex (8-week-old) were included in the study and were maintained under identical conditions of temperature (21 ± 1 °C), humidity (60 ± 5%), and light/dark cycle of 12 h and had free access to water and normal mouse chow diet. One month before either sham or TAC surgery, mice generated by different mothers were placed in cages (groups of 2–3 mice/cage). After surgery, mice were kept in single cages until study termination.

### Mouse model of pressure overload-induced cardiac hypertrophy and heart failure

Pressure overload was induced in adult C57BL/6 mice by TAC as previously described^40^. Mice were anesthetized by administration of 5% sevoflurane and 95% O_2_ and a suture was surgically placed across the aortic arch between the left common carotid artery and the innominate artery. Another group of animals underwent a left thoracotomy without aortic constriction (sham). Mice from sham and TAC groups were sacrificed 1 week (1w) and four weeks (4w) after surgery to perform molecular analyses. Only TAC animals with systolic pressure gradients > 40 mmHg measured by Doppler echocardiography were included in the study (see below).

### Cardiovascular ultrasound and Doppler

Cardiac function was non-invasively monitored by transthoracic echocardiography, using the Vevo 2100 high-resolution imaging system (Visual-Sonics, Toronto, ON, Canada) before, 1w and 4w after surgery as previously described^41^. Briefly, mice were anesthetized by an intraperitoneal injection of 0.1 ml/kg of mixture of 50% tiletamine and 50% zolazepam (Zoletil 100) and echocardiography was performed. Echocardiographic measurements were obtained from grayscale M-mode images at the mid-papillary level in the parasternal short-axis. Conventional measurements of the left ventricle (LV) included: left ventricular end-diastolic diameter (LVEDd), left ventricular end-systolic diameter (LVESd), interventricular septum end-diastolic diameter (IVSd), posterior wall end-diastolic diameter (PWd), heart rate (HR), % fractional shortening (FS %) and % ejection fraction (EF%). Pressure gradients across transverse aorta induced by TAC were evaluated using pulsed wave (PW) Doppler analysis 1w and 4w after surgery. Abdominal aortic flow was non-invasively estimated by PW Doppler before, 1, 3, 7, and 28 days after TAC or sham surgery.

### Microbiota sequencing and data analysis

To study fecal microbiota composition of sham and TAC mice, 16S rRNA gene sequences were obtained and pre-processed as previously described^42^. Resulting data were analyzed using Quantitative Insights Into Microbial Ecology (QIIME, version 1.9.1)^43^. Microbiota reads were collapsed into operational taxonomic units (OTUs) using a closed reference-based OTU picking method against Greengenes 16S gene database (GG, may 2013 version)^44^ at 97% of sequences similarity; picked OTUs were classified at different taxonomic levels with the GG database. Species classification was performed using SPecies IdentificatioN of metaGenOmic amplicons program (SPINGO version 1.3) with default parameters on a representative sequence of each OTU according to Ribosomal Database Project taxonomy and a 80% minimum bootstrap cutoff^45^. To avoid sample size biases in subsequent analyses, a sequence rarefaction procedure was applied using a depth of 26,396 sequences/sample. Species heterogeneity within each sample was assessed by employing two Alpha diversity metrics (the number Observed species and the Shannon entropy) and compared using a two-sample permutation t-test, using 999 Monte Carlo permutations to compute p-values. OTUs diversity among microbial communities (beta diversity) was assessed by calculating unweighted and weighted Unifrac distances and compared by using ANOSIM method with 999 permutations. Statistical differences in OTUs frequencies among groups across different taxonomic levels were assessed with Linear Discriminant Analysis (LDA) combined with effect size (LEfSe; p < 0.05 by Kruskal–Wallis test, p < 0.05 by pairwise Wilcoxon test and logarithmic LDA score of 2.0)^46^. Normalized OTU table, corrected for multiple 16S rRNA gene copy number, was used for metagenomes prediction in PICRUSt^47^. Kyoto encyclopedia of genes and genomes (KEGG) ortholog abundances of key functions involved in butyrate metabolism among groups were compared using two-tailed nonparametric Kruskal–Wallis test.

### RNA extraction and real-time semi-quantitative PCR

Mice colons were dissected at 1w and 4w after sham and TAC procedures for mRNA expression analysis^42^. Total RNA was extracted from colonic tissues by TRIzol Reagent (Bio-Rad Laboratories, Hercules, CA, USA), following the instructions of RNA extraction kit (NucleoSpin, MACHEREY–NAGEL GmbH & Co, Düren, Germany). cDNA was obtained using High-Capacity cDNA Reverse Transcription Kit (Applied Biosystems, Foster City, CA, USA) from 4 μg total RNA. PCRs were performed with a Bio-Rad CFX96 Connect Real-time PCR System instrument and software (Bio-Rad Laboratories). The PCR conditions were 15 min at 95 °C followed by 40 cycles of two-step PCR denaturation at 94 °C for 15 s, annealing extension at 55 °C for 30 s and extension at 72 °C for 30 s. Each sample contained 500 ng cDNA in 2X QuantiTect SYBRGreen PCR Master Mix and primers pairs to amplify interleukin-10 (*Il10*), zonuline-1 (*Tjp1*), occludin (*Ocln*) (Qiagen, Hilden, Germany) in a final volume of 50 μl. The relative amount of each studied mRNA was normalized to GAPDH as housekeeping gene, and data were analysed according to the 2^−ΔΔCT^ method.

### Serum ELISA assay

Blood samples were collected at sacrifice. Levels of IL-10, interleukin-1α (IL-1α), interleukin-6 (IL-6) and tumor necrosis factor α (TNFα) were measured using available ELISA kits (Thermo Scientific, Rockford, IL RBMS627R and RBMS629R, Biovendor R and D, Brno, Czech Republic). Lipopolysaccharide (LPS) levels were measured using the Limulus amebocyte lysate (LAL QCL-1000; Lonza Group Ltd., Basel, Switzerland), according to the manufacturer’s instructions.

### In vivo intestinal permeability assay

This examination is based on the intestinal permeability to fluorescein isothiocyanate-labeled dextran D4000 (FITC-dextran D4000) (Sigma-Aldrich, Milan, Italy) as previously described^42,48^. After 12 h of food withdrawal, mice were orally administered with FITC-dextran D4000 (50 mg/100 g body weight, 125 mg/ml). After 15 min, 1 h, 2 h and 4 h hours blood of all animals was collected by intracardiac puncture and centrifuged at 4 °C, 1200 rcf, for 10 min. Then plasma FITC-dextran concentration was determined (excitation, 485 nm; emission, 535 nm; HTS-7000 Plus-plate-reader; Perkin Elmer, Wellesley, MA, USA), using a standard curve generated by serial dilution of the tracer.

### Histological analysis

Colon samples from sham and TAC mice were collected, washed and placed in 10% neutral buffered formalin and embedded in paraffin wax; sections were cut and stained with haematoxylin and eosin (H&E; Carlo Erba, Italy). Sections were examined and scored using a 0-to-4 scale as previously described^42^. Histological scoring system was as follows: (a) the severity of inflammatory cell infiltration was evaluated by percentage of leukocyte density in lamina propria area and estimated in a high-power field (HPF) representative of the section (0 for no signs of inflammation, 1 for minimal < 10%, 2 for mild 10–25%, 3 for moderate 26–50%, 4 for marked > 51% with dense infiltrate); (b) the extent of the inflammation was estimated as expansion of leukocyte infiltration (0 for none, 1 for mucosal, 2 for mucosal and submucosal and 3 for mucosal, submucosal and transmural level); (c) the presence of fibrosis (0 for none, 1 if present).

For immunohistochemistry, 4-µm-thick sections were processed with the MACH1 Universal HRP Polymer Detection Kit (Biocare Medical LLC, Concord, CA). Colon tissues were mounted on positively charged glass slides (Bio-Optica, Milan). Antigen retrieval pretreatments were performed using a heat-induced epitope retrieval (HIER) citrate buffer pH6.0 (Bio- Optica, Milan, Italy) for 20 min at 98 °C; peroxide block was applied for 15 min at room temperature, and then the sections were incubated for 30 min with background sniper (Biocare Medical LLC). The primary antibodies were diluted in phosphate-buffered saline (PBS) and incubated overnight at 4 °C. Horseradish peroxidase (HRP) polymer was added for 30 min at room temperature. After every step, the sections were washed in 0.01 M PBS (pH 7.2–7.4). The reaction was revealed by using 3,30-diaminobenzidine (DAB) chromogen diluted in DAB substrate buffer. Finally, sections were counterstained in Carazzi’s haematoxylin (code n. 05-06012/L, Bio-Optica, Milan, Italy). Primary antibodies included rabbit monoclonal to ZO-1 (61-7300 ThermoFisher Scientific, Waltham, MA) diluted 1:100^49^.

### Protein extraction and immunoblotting

Colon samples were homogenized in a buffer containing 150 mmol/L NaCl, 50 mmol/L Tris–HCl (pH 8.5), 2 mmol/L EDTA, 1% v/v NP-40, 0.5% w/v deoxycholate, 10 mmol/L NaF, 10 mM sodium pyrophosphate, 2 mmol/L PMSF, 2 heart leupeptin, 2 heart aprotinin, pH 7.4, using the program Protein_1 on a GentleMACS tissue Dissociator (Miltenyi Biotec)^50^. Protein concentration in all lysates was measured by using a dye-binding protein assay kit (Bio-Rad) and a SmartSpec Plus spectrophotometer (Bio-Rad) reading at a wavelength of 595 nm. Immunoblotting was performed by using commercially available antibodies: anti-HIF-1α (mouse monoclonal, Novus Biologicals) and anti-tubulin (mouse monoclonal, Sigma-Aldrich). Secondary antibodies were purchased from Santa Cruz Biotechnology. Bands were visualized by enhanced chemiluminescence (ECL; Millipore Corporation) according to the manufacturer’s instructions, and were quantified by using densitometry (Chemidoc, Bio-Rad). Each experiment and densitometric quantification was separately repeated at least three times.

### Statistical analysis

All data presented are representative of three or more independent experiments and are expressed as mean ± SEM. All statistical analyses, with the exception of microbiota data processing, were conducted with Prism 8 statistical software; a minimum value of p < 0.05 was considered statistically significant. Comparisons between two groups were performed using the unpaired Student’s t-test. For experiments including three or more experimental groups, comparisons were made by one-way analysis of variance or 2-way ANOVA. Correction for multiple comparisons was made using the Tukey’s multiple comparison test. For each experiment, the number of mice used was specified in the figure legends.

## Supplementary Information

Supplementary information.
