# Supplementary material for: Transverse aortic constriction induces gut barrier alterations, microbiota remodeling and systemic inflammation
Source: Sci Rep. 2021 Apr 1;11:7404. doi: 10.1038/s41598-021-86651-y (PMC8016915; doi:10.1038/s41598-021-86651-y)

**Transverse Aortic Constriction Induces Gut Barrier Alterations, Microbiota Remodeling and Systemic Inflammation**

Nicola Boccella^1*^, Roberta Paolillo^1*^, Lorena Coretti^2,3,4,5*^, Stefania D’Apice^1*^, Adriano Lama^3,5^, Giuseppe Giugliano^1^, Gabriele Giacomo Schiattarella^1^, Mariella Cuomo ^6^, Ilaria d’Aquino^7^, Gina Cavaliere^8^, Orlando Paciello^7,5^, Maria Pina Mollica^5,8^, Giuseppina Mattace Raso^3,5^, Giovanni Esposito^1^, Francesca Lembo^3,5#^, Cinzia Perrino^1#^

*^1^Department of Advanced Biomedical Sciences, Federico II University, Naples, Italy.*

*^2^Fondazione Umberto Veronesi, Milan, Italy.*

*^3^ Department of Pharmacy, Federico II University, Naples, Italy.*

*^4^Department of Physiology and Biochemistry, Faculty of Medicine and Surgery, University of Malta, Msida, Malta.*

*^5^Task Force on Microbiome Studies, Federico II University, Naples, Italy.*

*^6^Department of Molecular Medicine and Medical Biotechnology, Federico II University, Naples, Italy.*

*^7^Department of Veterinary Medicine and Animal Productions, Unit of Pathology, Federico II University, Naples, Italy.*

*^8^Department of Biology, Federico II University, Naples, Italy.*

*These authors equally contributed to this work

**^#^**These authors equally contributed to this work

**Address correspondence to:**

Cinzia Perrino MD PhD FESC, Federico II University, Dept. of Advanced Biomedical Sciences

Via Pansini 5, 80131, Naples, Italy, tel: +39 0817462234, fax: +39 0817462223, email: [perrino@unina.it](mailto:perrino@unina.it)

Francesca Lembo PhD, Federico II University, Dept. of Pharmacy, Via D. Montesano 49, 80131, Naples, Italy, tel: +39 081678651, email: [frlembo@unina.it](mailto:frlembo@unina.it)

**Supplementary Figure 1 legend**

**HIF1-α levels in colon samples of sham e TAC mice 1w and 4w after procedure**

Representative immunoblot (top) and densitometric (bottom) analysis of HIF1-α protein levels in colon samples. Tubulin was used as protein loading control (∗p < 0.05).

**Supplementary Figure 2 legend**

**Intestinal barrier integrity and inflammation in pre-surgery and sham (1w and 4w) mice.**

mRNA levels of *Tjp1*, *Ocln* and *Il-10* in colon samples from pre-surgery, sham 1w and sham 4w mice.

**Supplementary information on cropped Hif1- α /Tubulin Figure S1**


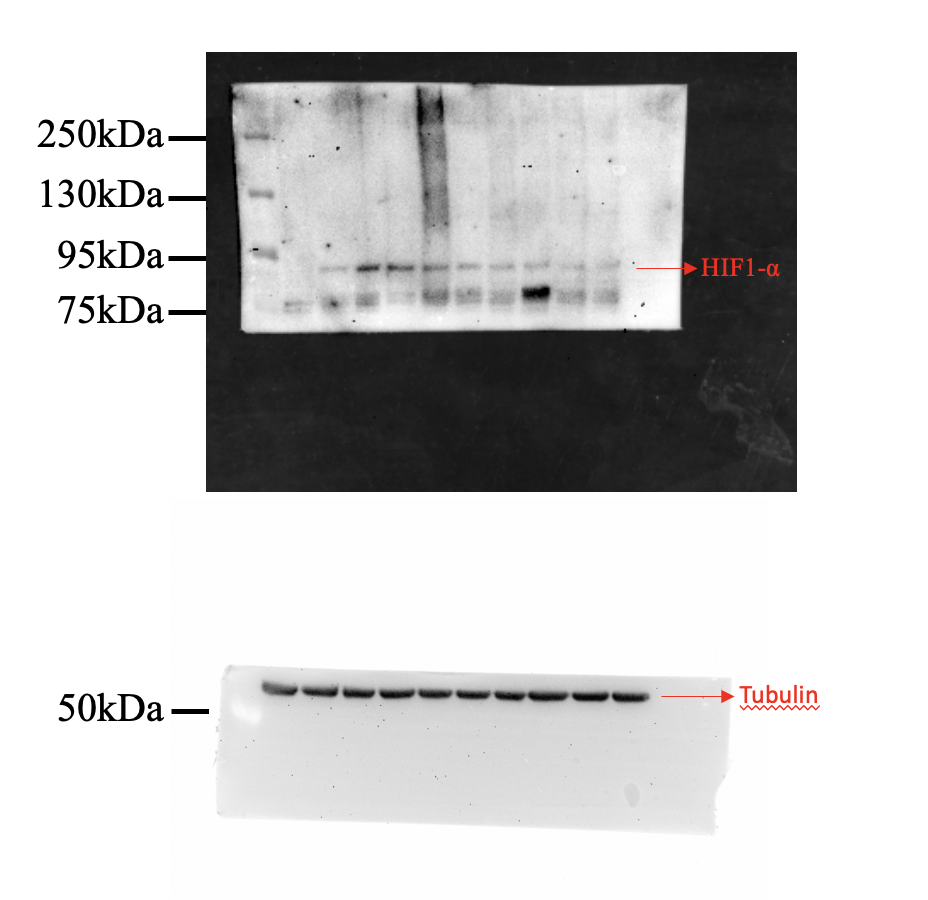

Supplement: Supplementary file 1 — Supplementary information. [file 41598_2021_86651_MOESM1_ESM.docx]
